# A solute-binding protein for iron transport in *Streptococcus iniae*

**DOI:** 10.1186/1471-2180-10-309

**Published:** 2010-12-01

**Authors:** Lili Zou, Jun Wang, Baofeng Huang, Mingquan Xie, Anxing Li

**Affiliations:** 1 Key Laboratory for Aquatic Products Safety of Ministry of Education/State Key Laboratory of Bio-control, School of Life Sciences, Sun Yat-sen University, 135 Xingang West Road, Haizhu District, Guangzhou 510275, PR China

## Abstract

**Background:**

*Streptococcus iniae *(*S. iniae*) is a major pathogen that causes considerable morbidity and mortality in cultured fish worldwide. The pathogen's ability to adapt to the host affects the extent of infection, hence understanding the mechanisms by which *S. iniae *overcomes physiological stresses during infection will help to identify potential virulence determinants of streptococcal infection. Grow *S. iniae *under iron-restricted conditions is one approach for identifying host-specific protein expression. Iron plays an important role in many biological processes but it has low solubility under physiological condition. Many microorganisms have been shown to be able to circumvent this nutritional limitation by forming direct contacts with iron-containing proteins through ATP-binding cassette (ABC) transporters. The ABC transporter superfamilies constitute many different systems that are widespread among living organisms with different functions, such as ligands translocation, mRNA translation, and DNA repair.

**Results:**

An ABC transporter system, named as *mtsABC *(metal transport system) was cloned from *S. iniae *HD-1, and was found to be involved in heme utilization. *mtsABC *is cotranscribed by three downstream genes, i.e., *mtsA*, *mtsB*, and *mtsC*. In this study, we cloned the first gene of the *mtsABC *transporter system (*mtsA*), and purified the corresponding recombinant protein MtsA. The analysis indicated that MtsA is a putative lipoprotein which binds to heme that can serve as an iron source for the microorganism, and is expressed *in vivo *during Kunming mice infection by *S. iniae *HD-1.

**Conclusions:**

This is believed to be the first report on the cloning the ABC transporter lipoprotein from *S. iniae *genomic DNA. Together, our data suggested that MtsA is associated with heme, and is expressed *in vivo *during Kunming mice infection by *S. iniae *HD-1 which indicated that it can be a potential candidate for *S. iniae *subunit vaccine.

## Background

*Streptococcus iniae *(*S. iniae*) is a hemolytic Gram-positive coccus that is a major pathogen of culture fish. It has been associated with disease outbreak in several species of freshwater and marine fish cultured worldwide, including tilapia [1,2], barramundi [3], channel catfish [4], hybrid striped bass [1,5], Japanese flounder [6,7], olive flounder [8], rabbitfish [9], and rainbow trout [9,10]. Streptococcal infection can lead to serious symptoms such as meningoencephalitis and generalized septicaemia with high mortality rates of up to 50% [9,11]. *S. iniae *is also known to be an opportunistic pathogen that can cause fulminant soft tissue infection in humans, such as bacteremic cellulitis, septicarthritis, and endocarditis [12]. Identifying potential virulence determinants of streptococcal infection will eventually help to the control and eradication of the disease.

Iron plays a significant role in many biological processes and is vital for several metabolic processes. Moreover, many proteins such as cytochromes and tricarboxylic acid metalloenzymes use iron as a cofactor [13]. Iron is also required for important cellular functions such as the transport and storage of oxygen and as a catalyst in electron transport processes [14]. The levels of several virulence determinants produced by bacterial pathogens, such as toxins and hemolysins, are depressed under iron-restricted conditions [15]. Despite its abundance in the natural environment, iron has low solubility under physiological conditions. Moreover, it may be associated with heme or hemo-proteins such as transferrin, lactoferrin, haptoglobin, hemoglobin, and ferritin and such forms do not readily support the growth of microorganisms. Many microorganisms circumvent this nutritional limitation by forming direct contacts with iron-containing proteins through ATP-binding cassette (ABC) transporters.

The ABC transporter superfamilies constitute many different systems that are widespread among living organisms and show different functions, such as ligands translocation, mRNA translation, and DNA repair. The general principle of ABC transport systems involves the ligands translocation through a pore formed by two integral membrane protein domains. This is accompanied by ATP hydrolysis through two nucleotide-binding domains associated with the cytoplasmic side of the pore. In bacteria, ligand translocation is preceded by interaction with an accessory component, i.e., the periplasmic-binding protein [16].

In this study, an ABC transporter member, named as *mtsABC *(metal ABC transport system) was cloned from *S. iniae *HD-1 which is cotranscribed by three genes and was shown to share amino acid sequence homology with the metal ABC transport proteins of other Gram-positive and Gram-negative bacteria. BLAST-mediated sequences similarity searches of the derived amino acid sequences of the *mtsABC *operon indicated that *mtsA *encodes a metal solute-binding lipoprotein, *mtsB *encodes an ATP-binding protein (ATPase), and *mtsC *encodes a transmembrane permease protein. Our data showed that MtsA is a lipoprotein, and associated with heme. Moreover, this protein is expressed *in vivo *during Kunming mice infection by *S. iniae *HD-1. These results provide information on the role of MtsA in heme utilization and the possibility of using MtsA as an effective *S. iniae *vaccine candidate.

## Results

### Cloning and reverse transcriptase-PCR analysis of *mtsABC*

**Figure 1:**
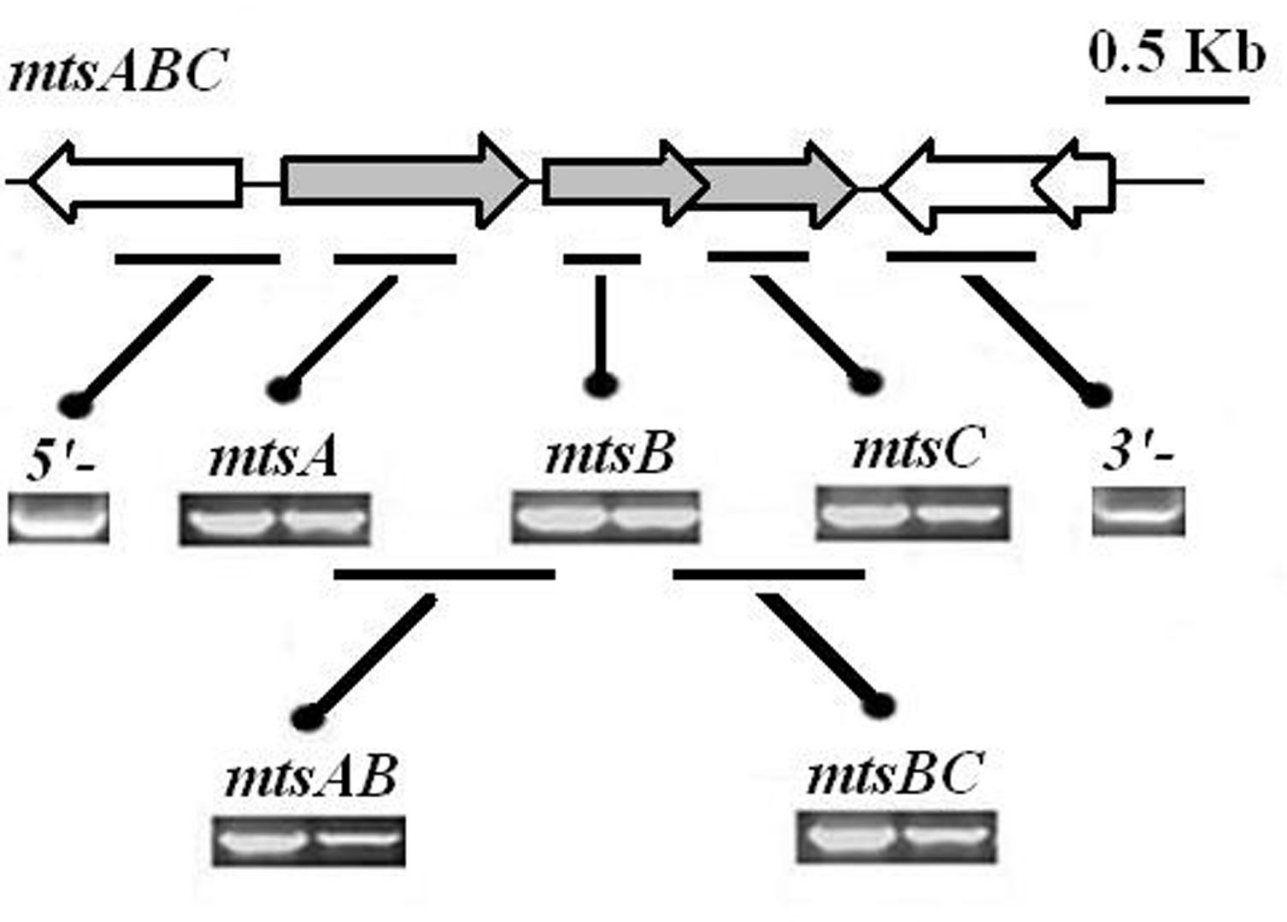
**Reverse transcriptase-PCR analysis demonstrates a polycistronic transcript of *mtsABC *mRNA**. Total RNA from *S. iniae *HD-1 was reverse transcribed into cDNA, and PCR was performed with ORF-specific primers. Each box contains products with the same primer pairs. For PCR, *S. iniae *HD-1 genomic DNA was used as the template (on the left), and for reverse transcriptase-PCR, *S. iniae *HD-1 RNA was used as the template (on the right).

To clone *mtsABC *from *S. iniae *HD-1, primers designed based on the conserved regions of the published amino acid sequence of metal ABC transporter were used. The PCR products from genomic DNA template were subsequently sequenced by Invitrogen Corporation. The results showed that the ORFs of *mtsA *[GeneBank: HQ170628], *mtsB *[GeneBank: HQ170629], *mtsC *[GeneBank: HQ170630], *mtsAB*, and *mtsBC *had 930, 729, 852, 1724, and 1574 bp respectively. Reverse transcriptase-PCR analysis confirmed that the *mtsA *gene is the first of three contiguous ORFs that are preceded by a potential promoter region. These three genes are transcribed in the same direction and have very short intergenic sequences, with transcription terminating between *mtsA *and *mtsB*, suggesting that these genes constitute a single transcriptional unit (Figure 1). No corresponding PCR products were obtained with the same mRNA sample as the template, indicating that the RNA sample was not contaminated with DNA.

### Sequence analysis of *mtsABC*

**Figure 2:**
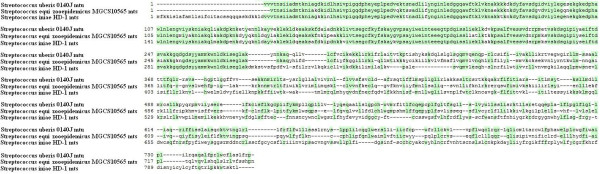
**Sequence alignment of MtsABC and its homologues**. The amino acid sequences were aligned using the the SECentral Align Multi 4 program. Dark shading represented identical amino acid residues.

ABC systems are widespread among living organisms and have been detected in all genera of the three kingdoms of life. These systems show remarkable conservation in the primary sequence of the cassette and in the organization of constitutive domains or subunits [17,18]. All ABC systems share a highly conserved ATP-hydrolyzing domain (nucleotide-binding domain [NBD]) that is unequivocally characterized by three short sequence motifs, i.e., Walker A, Walker B, and a signature motif that is unique to ABC proteins and is located upstream of the Walker B motif [19-24]. BLAST of the derived amino acid sequences of the *mtsABC *operon indicated that *mtsA *encodes a metal solute-binding lipoprotein (MtsA, 309 residues), *mtsB *encodes an ATP-binding protein (MtsB, 242 residues), and *mtsC *encodes a transmembrane permease protein (MtsC, 283 residues). The closest homologs for these proteins are putative metal ABC transporter proteins encoded by the *mtu *locus of *Streptococcus uberis *0140J and the *mts *locus of *Streptococcus equi *subsp. *zooepidemicus *MGCS10565 (Additional file 1, Table S1, and Figure 2). *mtsA *contains a helical backbone metal receptor (TroA-like domain) that functions in the ABC transport of ferric siderophores and metal ions such as Fe^3+^, Mn^2+^, Cu^2+^, and/or Zn^2+ ^(Additional file 1, Table S2). *mtsB *contains Walker site A, Walker site B, a signature sequence, and the 4^th ^motif as defined by Linton & Higgins [25]. *mtsC *contains eight transmembrane subunits (TMs) of the periplasmic-binding protein (PBP)-dependent ABC transporters that are possibly involved in the uptake of siderophores, heme, vitamin B_12_, or divalent cations (Additional file 1, Table S2). Based on these observations, we concluded that *mtsABC *is a member of the ABC transporter systems.

**Figure 3:**
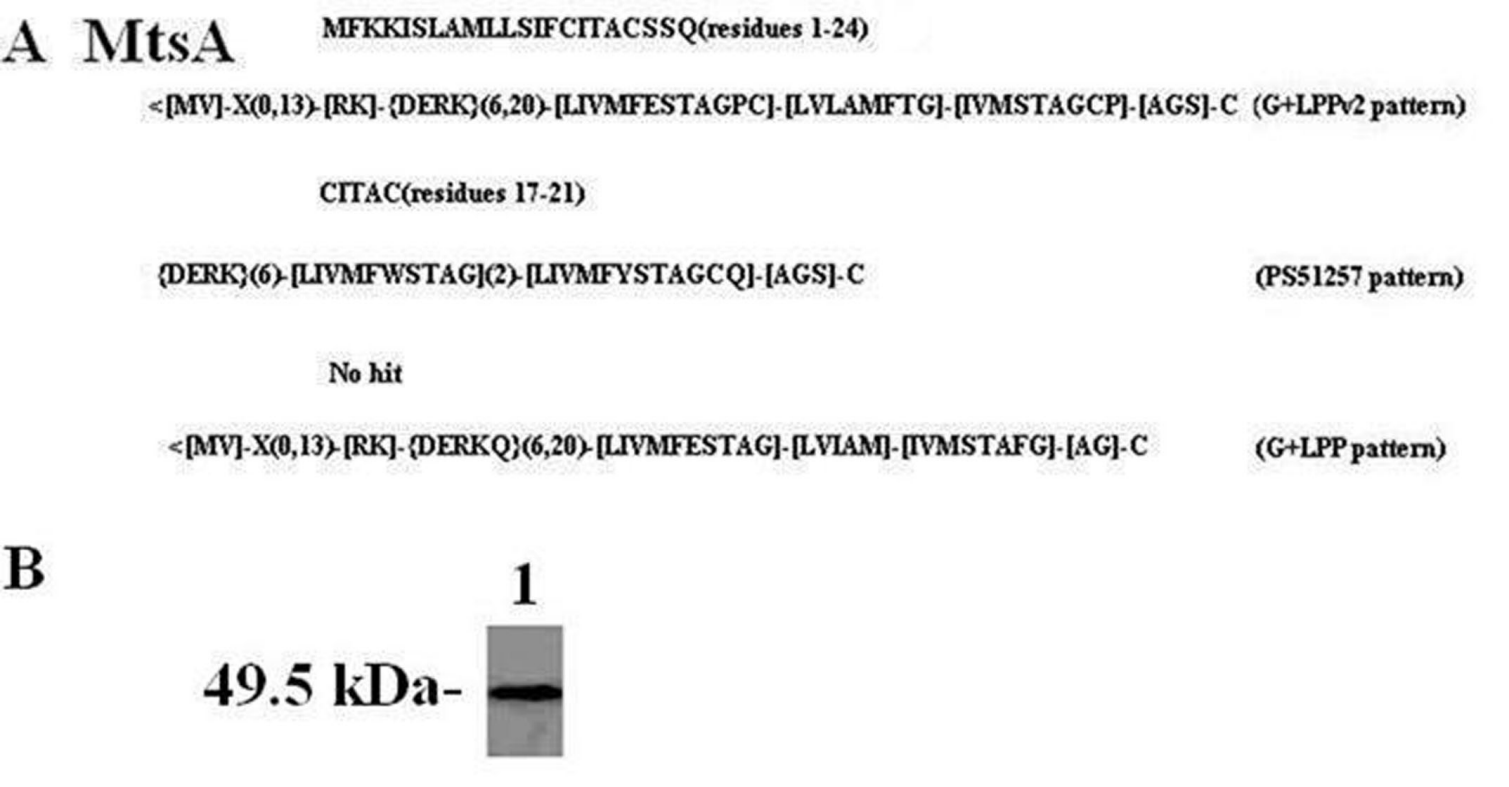
**Analysis of the lipoprotein sequence patterns of MtsA by ScanProsite and the western blotting**. (A) The *mtsABC *lipoprotein was assessed by ScanProsite. The results showed that amino acid residues D1 to D24 (MFKKISLAFAMLLSIFCITACSSQ) hit G+LPPv2 pattern, and amino acid residues D17 to D21 (CITAC) hit PS51257 pattern. The symbol "<" indicates that the pattern is restricted to the N terminus, and X is any amino acid. (B) Western blotting analysis results of the lipoproteins extracted with Triton X-114.

Three patterns of signal peptide (Additional file 1, Table S3) were used to identify bacterial lipoproteins from bioinformatics data [26]. To characterize the MtsA protein, the ScanProsite analysis was performed. The results indicated that the sequence of MtsA showed similarity to the following 2 patterns: residues D1 to D24 (MFKKISLAFAMLLSIFCITACSSQ) hit the G+LPPv2 pattern, and residues D17 to D21 (CITAC) hit the PS51257 pattern (Figure 3A) [27], which suggested that MtsA is a lipoprotein. *mtsA *contains an lipoprotein peptidase cleavage site signal sequence as defined by Linton & Higgins [25]. To confirm that MtsA is a lipoprotein, the crude cell lysate of *S. iniae *HD-1 was mixed with Triton X-114, and the detergent phase was analyzed by western blotting using rabbit anti-MtsA antibodies (Figure 3B). The results showed that MtsA protein was extracted by Triton X-114. Together, the results indicated that MtsA protein is a lipoprotein.

### Purification of recombinant MtsA

**Figure 4:**
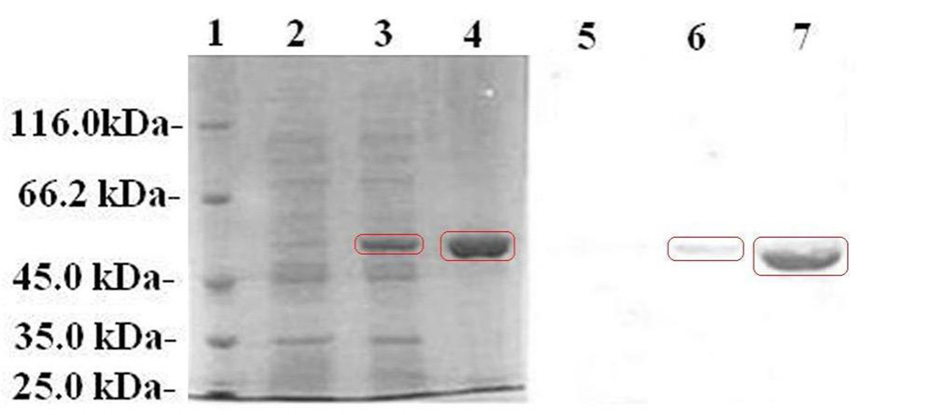
**SDS-PAGE and western blotting analysis of expressed and purified MtsA**. Lanes 1~4, SDS-PAGE showing the purification results of MtsA. The gels were stained with Coomassie brilliant blue. Lane 1, molecular mass marker; lane 2, *E. coli *with control pet-32a-c (+) vector; lane 3, *E. coli *lysate containing MtsA (approximately 49.5-kDa); lane 4, purified MtsA (approximately 49.5-kDa). Lanes 5~7, western blotting results of purified MtsA. Lane 5, *E. coli *with control pet-32a-c (+) vector; lane 6, *E. coli *lysate containing MtsA (approximately 49.5-kDa); lane 7, purified MtsA (approximately 49.5-kDa).

To be able to further characterize MtsA, we first expressed recombinant MtsA consisting of amino acid residues D27 to D310 that lacked the putative signal sequence. Briefly, *mtsA *gene was cloned and the PCR product was isolated from the plasmid after a double digestion with restriction enzymes *Bam*HI and *Xho*I, and ligated into the compatible site of pET-32a-c (+) Vector to yield recombinant protein MtsA. The expressed MtsA had a molecular mass of 49.5-kDa (Figure 4) with a tag from Trx·Tag to *EcoR V *of pET-32a-c (+), which has a molecular weight of 17.7-kDa. The expression level of MtsA peaked after induction with 1 mM IPTG at 37°C for 4 h. The MtsA protein was purified from *E. coli *BL21 (DE3) under native condition n the soluble form and immunized the New Zealand white rabbits. The results showed that the rabbit anti-MtsA antibody titers increased from essentially zero to 1:50,000 after four rounds of immunization (Additional file 1, Table S4). The western blotting analysis was performed to show the specificity of immunized sera against purified MtsA (Figure 4, and Additional file 2, Figure S3-4).

### Subcellular localization of MtsA

**Figure 5:**
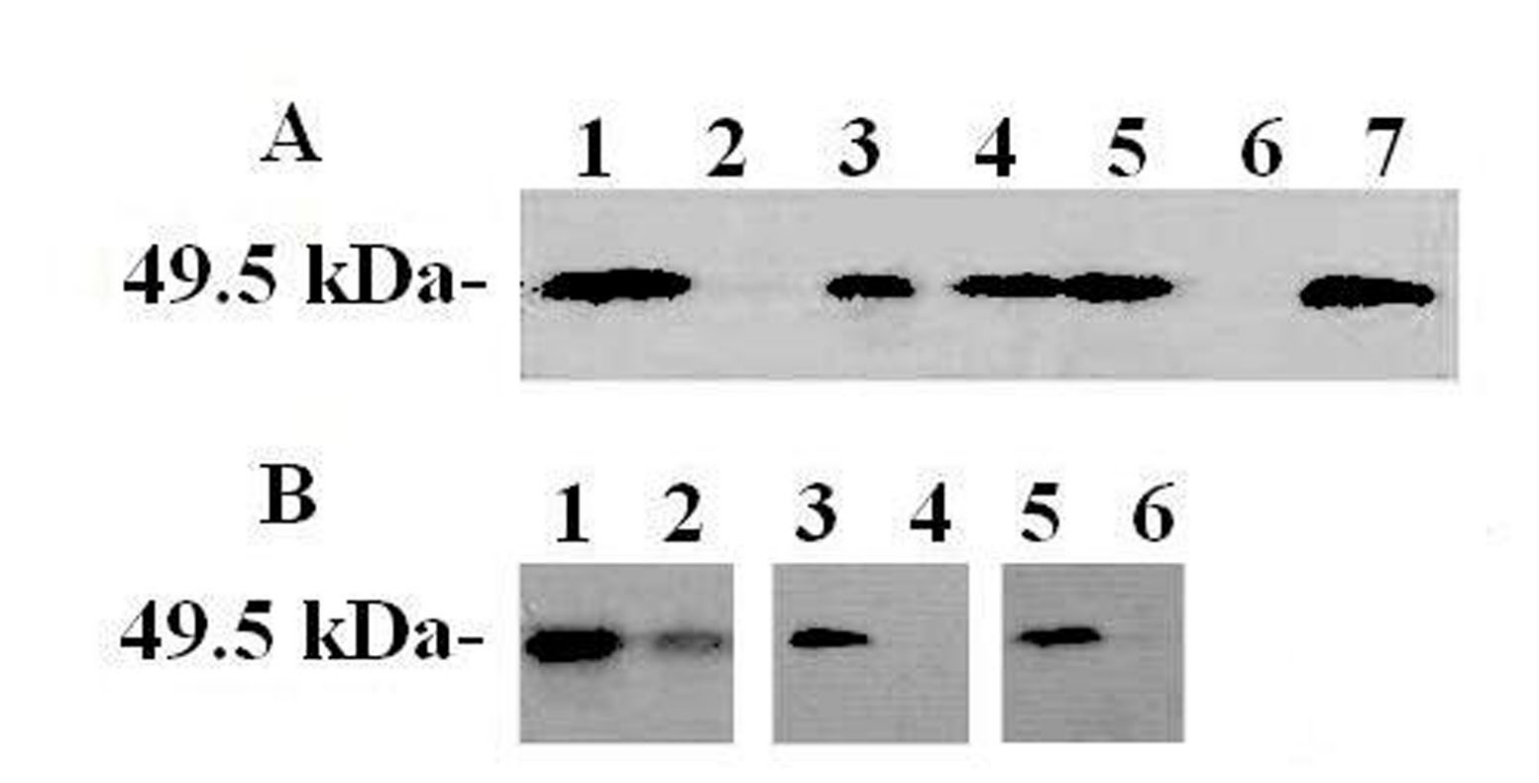
**Detection of the subcellular localization of MtsA in *S. iniae *HD-1 by western blotting**. (A) The cellular fractions of *S. iniae *HD-1 and rabbit anti-MtsA antibodies were used for the western-blot assay. Lane 1, *S. iniae *HD-1 lysate; lane 2, soluble fraction of cells; lane 3, particulate fraction of cells; lane 4, cell wall extracts; lane 5, protoplast; lane 6, particulate fraction of protoplasts; and lane 7, soluble fraction of protoplasts. (B) Surface exposure of MtsA. Cells (lanes 1 and 2), cell wall extracts (lanes 3 and 4), and protoplasts (lanes 5 and 6) of *S. iniae *HD-1 were treated with proteinase K and analyzed by western blotting. Lanes 1, 3 and 5 show the untreated control, while lanes 2, 4 and 6 show samples treated with proteinase K for 1 h.

To determine the subcellular localization of MtsA, the western blotting was performed with the cellular fractions of *S. iniae *HD-1 using rabbit anti-MtsA antibodies (Figure 5A). MtsA was detected in the particulate fraction of the cells when the cellular fractions were prepared by centrifugation of the crude cell lysate (the first treatment). MtsA was found to be associated with the protoplast and cell wall extracts when the cellular fractions were prepared by protoplast formation. After separation of the protoplasts, MtsA was detected in the particulate fraction (the second treatment).

To detect surface exposure of MtsA, cells of *S. iniae *HD-1 cells were harvested, washed, centrifuged, and resuspended in PBS. The cells were subjected to proteinase K (5 μg ml^-1^) treatment with gentle agitation at room temperature for 1 h, and the cells were collected. Western blotting showed that peptide fragments in the cells can be detected after 1 h incubation with proteinase K. However, when the cell wall extracts and protoplasts were used in the experiment, it were completely hydrolyzed and no peptide fragments were detected (Figure 5B). Together, this result indicated that MtsA is not exposure on surface, but is on the outside of the cytoplasmic membrane and is buried inside the cell wall.

### MtsA had heme-binding activity

**Figure 6:**
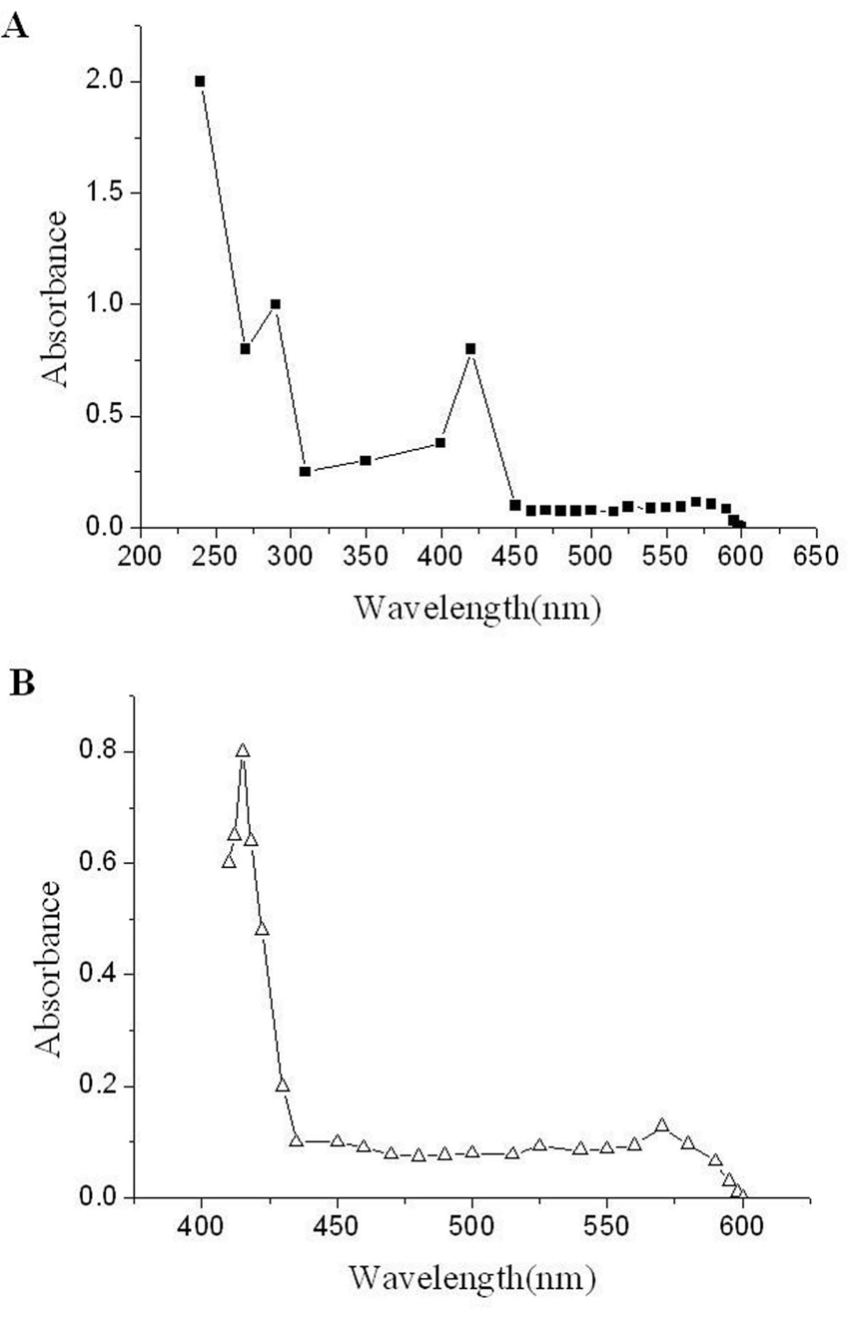
**Detection of the heme-binding activity of purified MtsA by the pyridine hemochrome assay**. (A) UV-visible absorption spectrum of 20 μM purified MtsA (■ line) in 50 mM Tris-HCl (pH 8.0). (B) UV-visible absorption spectrum of 20 μM purified KatG (Δ line) in 50 mM Tris-HCl (pH 8.0).

To examine whether heme is the chromophore associated with MtsA, the pyridine hemochrome assay was performed [28]. The UV-visible absorption spectrum of purified MtsA exhibited peaks at 275, 420, 525, and 560 nm, which were identical to those obtained from purified KatG, a well-known heme-containing protein with spectral peaks at 418, 524, and 556 nm. The molar ratio of associated heme to purified MtsA was 0.806 (Figure 6), this value is consistent with the hypothesis that one protein molecule is associated with one heme molecule.

To determine whether iron is present and its amount in purified MtsA, the ICP-AES analysis were performed. The results showed that Fe was present (Additional file 1, Table S5) in purified MtsA; however, four other bivalent metallic elements Ca, Mg, Zn and Mn were not detected. The amount of iron present in purified MtsA (20 μM) was 1.43, 1.38, and 1.33 mg L^-1^, in three independent purification experiments respectively.

### *In vivo *production of MtsA during *S. iniae *HD-1 infection

**Figure 7:**
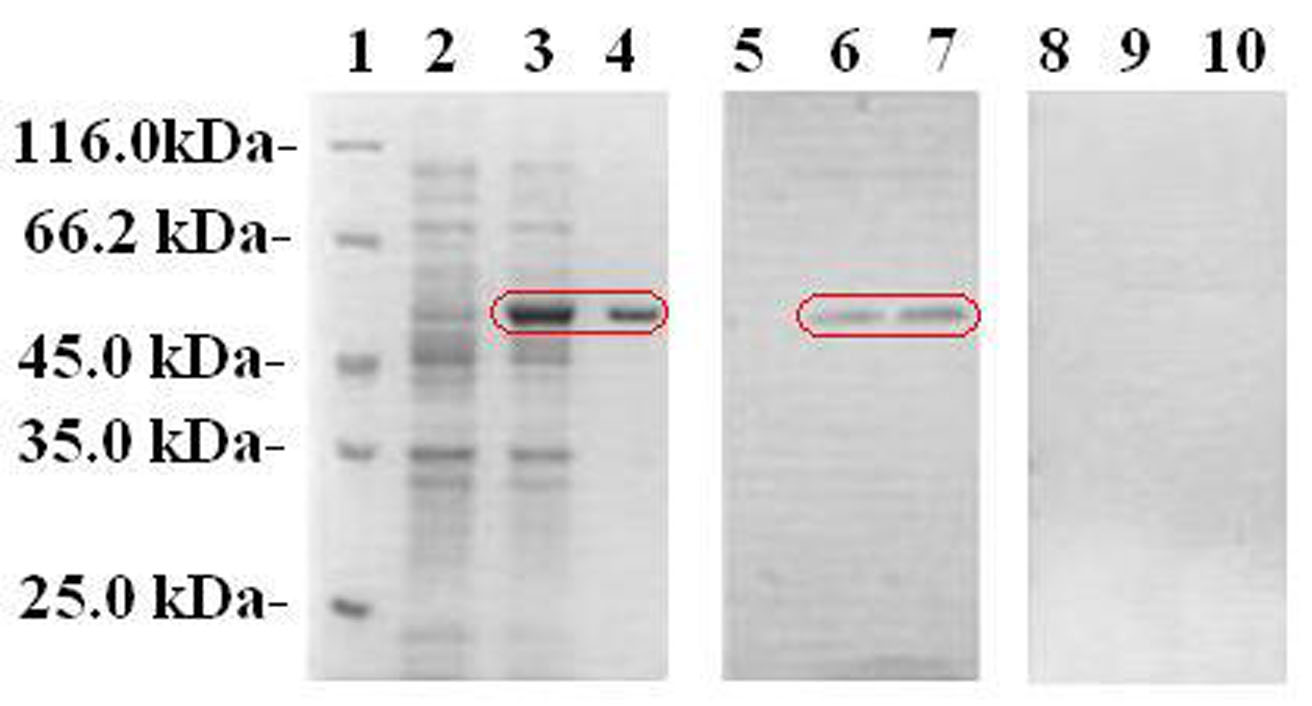
**Western blotting analysis of anti-MtsA antibodies in infected sera from Kunming mice with *S. iniae *HD-1 infection**. SDS-PAGE analysis showing the purification results of MtsA. The gel was transferred to a nitrocellulose membrane and blotted with infected sera from mice. The gels were stained with Coomassie brilliant blue. Lane 1, molecular mass marker; lane 2, *E. coli *with control pet-32a-c (+) vector; lane 3, *E. coli *lysate containing MtsA (approximately 49.5-kDa); lane 4, purified MtsA (approximately 49.5-kDa); lanes 5~7, western blot results of infected sera, lanes 8~10, western blot results of control sera; lanes 5 and 8, western blot results of *E. coli *with the control vector; lanes 6 and 9, *E. coli *lysate containing MtsA, and lanes 7 and 10, purified MtsA (approximately 49.5-kDa).

To determine whether MtsA is produced *in vivo *during *S. iniae *infection, we infected Kunming mice with *S. iniae *HD-1 and performed western blotting analysis with purified MtsA to determine the presence of anti-MtsA antibodies in infected sera (Figure 7). The results indicated that MtsA is produced *in vivo *during experimental *S. iniae *HD-1 infection.

## Discussion

Heme is an important nutrient for several bacteria and can serves as a source of essential iron. The most abundant source of iron in the body is heme, so it is not surprising to find that pathogenic bacteria can use heme as an iron source [29]. The presence of the central iron atom in heme allows it to undergo reversible oxidative change and act as a virulence-regulated determinant [30-36]. It is necessary for bacterial pathogens to acquire sufficient iron from their surroundings, and scavenging heme from the environment requires much less effort than synthesizing it *de novo *[30,34].

Acquiring iron from the micro-environment is important for the growth of bacterial pathogens. Pathogens often use low environmental iron levels as a signal to induce virulence genes [14]. Many pathogenic bacteria secrete exotoxins, proteases, and siderophores to rapidly increase the local concentration of free heme [37], and it is common for pathogens to directly acquire iron from host iron-binding proteins by using receptor-mediated transport systems specific for host-iron complexes [38].

To define the role of MtsA in heme utilization, the binding activity and subcellular localization of purified MtsA were investigated. The coding sequence of *mtsA *was cloned into the expression vector pet-32a-c (+). The major induced protein in *E. coli *(BL21) migrated as a 49.5-kDa band in SDS-PAGE gels; this size is consistent with the predicted molecular mass of MtsA. The 49.5-kDa MtsA was purified by Ni^2+ ^affinity chromatography and reacted with anti-MtsA antibodies from infected mice to confirm the *in vivo *production of MtsA. The UV-visible absorbance spectrum of KatG is a typical heme-containing protein, and the results of the pyridine hemochrome assay indicated that MtsA is associated with heme. Moreover, measurements of the iron level by ICP-AES indicated that purified MtsA is a holo-protein that is associated with iron.

In general, there are four major types of cell surface display proteins in Gram-positive bacteria, which are as follows: proteins anchored to the cytoplasmic membrane by hydrophobic transmembrane domains; lipoproteins that are covalently attached to membrane lipids after cleavage by signal peptides II; proteins that contain the C-terminal LPXTG-like motif and are covalently attached to peptidoglycan by sortase; and proteins that recognize some cell wall components by specific domains [39]. ABC transporters are integral membrane proteins that transport diverse substrates across lipid bilayers [40]. In bacteria, ABC transporters catalyze the uptake of essential nutrients or the extrusion of toxic substances [41]. ABC importers, present only in prokaryotes, require a binding protein that delivers the captured substrate to the external face of the transporter [42]. As MtsA is a solute-binding protein of the ABC transporter, its major function is presumed to be the capture and transfer of iron compounds to the downstream gene of the iron transport system of *S. iniae *HD-1. The signal peptide pattern analysis and Triton X-114 extraction results confirmed that MtsA is a lipoprotein. This result is reliable because the original G+LPP pattern was present in the analysis of the signal peptide features of 33 experimentally verified lipoproteins. Lipoproteins in Gram-positive bacteria are cell envelope proteins anchored to the outer leaflet of the plasma membrane. Lipid modification is achieved through covalent addition of a diacylglyceride to an indispensable cysteine residue in the lipoprotein signal peptide that provides a common anchoring mechanism for what is now recognized as an abundant and functionally diverse class of peripheral membrane proteins [40]. In Gram-positive bacteria, substrate-binding proteins of ABC transporters are typically lipoproteins [41,42], and the western blotting results is consistent with the notion that MtsA is an ABC transporter lipoprotein [43,44].

The results of this study indicated that *mtsABC *is a member of the ABC transporter family. MtsA protein is a solute-binding protein that can bind to heme and facilitate the latter's use as a substrate by the *S. iniae*. Western blotting indicated that MtsA is produced *in vivo *during experimental *S. iniae *HD-1 infection, and MtsA may be a potentially useful *S. iniae *protein vaccine candidate. Further studies are needed to clarify the role of MtsA in the utilization of other iron compounds and other important cations, and to establish its candidacy as a useful *S. iniae *vaccine component.

## Conclusions

In summary, this study presents MtsA as a novel solute-binding protein that can contribute to iron transport. This is the first ABC transporter member to be identified from *S. iniae*. We have shown that MtsA is a lipoprotein which can bind to heme, and is expressed *in vivo *during Kunming mice infection by *S. iniae *HD-1. More importantly, this is the first report on the cloning of ABC transporter lipoprotein from *S. iniae *genomic DNA, and its immunogenicity is indicative of its possible use as an *S. iniae *subunit vaccine.

## Methods

### Bacterial strains and growth conditions

*Streptococcus iniae *HD-1 was isolated from Threeband sweetlips (*Plectorhynchus cinctus*) from Guangdong province, PRC. The microorganism was stored in our lab and cultured according to the methods described by Zhou *et al *[45]. Briefly, *S. iniae *isolate HD-1 cells were grown in brain heart infusion broth (BHI, Oxoid Ltd.), and BHI broth with 1.5% agar (Guangdong Huankai Microbial Sci. & Tech, Co., Ltd.) was used as the solid medium. *Escherichia coli *DH5α and BL21 (DE3) strains (Beijing Newprobe Biotechnology Co., Ltd.) were used for gene cloning and protein expression, respectively.

### Cloning and reverse transcription analysis of *mtsABC*

Genomic DNA was extracted from the *S. iniae *HD-1 strain using the Wizard genomic DNA purification kit (Promega Co., Ltd.), as recommended by the manufacturer, and the material was quantified by measuring the absorbance at 260 nm. PCR was carried out with 1 μg of DNA using the primers listed in Additional file 1, Table S6. The primers were designed based on the conserved regions of the published amino acid sequence of metal ABC transporter (Additional file 1, Table S6-1), and the full-length product was obtained by SiteFinding-PCR (Additional file 1, Table S6-2, 6-3), as described by Tai *et al *[46]. The PCR products were sequenced to rule out spurious mutations (Invitrogen Co., Ltd.).

*S. iniae *HD-1 cells grow to the logarithmic phase were harvested by centrifugation, and total RNA was extracted by the Pure Yield™ RNA midiprep system (Promega, USA, Co., Ltd.). Total RNA was then incubated with RNase I at 37°C for 30 min to remove the contaminating genomic DNA. The material was quantified spectrophotometrically by ultraviolet absorption spectrometry (CE2302, Gene Quest), and its integrity was verified on a 0.8% agarose gel. First-strand cDNA was synthesized from 1 μg total RNA using the first-strand cDNA synthesis kit with ReverTra Ace-α-reverse transcriptase (Toyobo Co., Ltd.). The cDNA synthesized above was used as the template to amplify genes using the ORF-specific primers listed in Additional file 1, Table S7, and the PCR products were sequenced at Invitrogen Corporation to confirm their specificity.

### Expression of recombinant MtsA

The genomic DNA of *S. iniae *HD-1 was used as the template to amplify the gene encoding amino acids 27 to 310 of *mtsA *together with primers 5'-GCGGGATCCGCCTCTAAAGATAAG-3' (underlined nucleotides refer to the *Bam*HI restriction site-initiation codon) and 5'-GCGCTCGAGTTATTTTGCTAAGCCTTCTGAA-3' (underlined nucleotides refer to the *Xho*I restriction site-stop codon), which were designed from the sequenced *mtsA *gene. The protein from this cloned amino acid sequence lacks the presumed signal sequence (amino acids 1 to 26). The cloned amino acid fragment was sequenced by Invitrogen Corporation to rule out the possibility of spurious mutations. Recombinant MtsA was purified from *E. coli *BL21 (DE3) under native conditions using nickel-nitrilotriacetic acid (Ni-NTA) columns (Qiangen, USA) as recommended by the manufacturer. The protein purified by this protocol was free of contaminating proteins, as assessed by sodium dodecyl sulfate-polyacrylamide gel electrophoresis (SDS-PAGE). It was quantified by the Bradford assay (CE2302, Gene Quest) using BSA (0.5 mg ml^-1^) as the standard. Specific fractions were then pooled.

### Preparation of anti-MtsA antibodies

Anti-sera against histidine-tagged MtsA were prepared in male New Zealand white rabbits (2.2 kg), and approval from the Animal Ethics Committee of Life Sciences Institute was obtained prior to using the animals for research. The experiments were performed as stipulated by the China State Science and Technology Commission [47]. Rabbits were purchased from Guangdong Laboratory Animals Research Center and acclimatized for 2 weeks in the laboratory of the Life Science Institute prior to use. The rabbits were maintained at the SPF animal center and fed twice daily. They were immunized with 850 μg purified MtsA in 100 μl complete Freund adjuvant (Sigma-Aldrich, Inc.) and then boosted with 170 μg MtsA in 100 μl incomplete Freund adjuvant (Sigma-Aldrich, Inc.) three times at an interval of 15 days. The sera were collected 1 day before the first immunization and 7 days after each booster dose. Purified MtsA and collected sera were used to determine the rabbit anti-MtsA antibody titer by the dot blotting assay.

### Extraction of the *S. iniae *HD-1 lipoprotein

TritonX-114 was used to extract the *S. iniae *HD-1 lipoprotein, according to the method modified by Cockayne *et al *[48,49]. Briefly, *S. iniae *HD-1 cells were cultured, harvested, suspended, and sonicated. Next, 100 μl of 10% TritonX-114 in PBS was added to 2 ml of HD-1 cells lysate and incubated at 4°C for 2 h. After centrifugation at 13,000 × *g *for 10 min, the supernatant was transferred to a fresh tube and incubated at 37°C for 30 min to allow phase separation. The detergent layer was retained after centrifugation at 13,000 × *g *for 10 min at room temperature, washed with 1 ml PBS at 4°C for 1 h, and separated from the aqueous phase after incubation at 37°C [50]. The detergent layer was diluted 1:1 with water, and analyzed by western blotting using the rabbits anti-MtsA antibodies.

### Preparation of MtsA cellular fractions

To determine the subcellular localization of MtsA in *S. iniae *HD-1, the cellular fractions were subjected to two different treatments. In the first treatment procedure, *S. iniae *HD-1 cells were cultured overnight in 50 ml BHI, harvested, and resuspended in one-tenth volume of Tris buffer (1 M, pH 7.4), and disrupted by sonication (300 W, 5 min). After removing unbroken cells by centrifugation at 10,000 × *g*, the crude cell lysate was further centrifuged at 248,000 × *g *for 1 h (Optima™L-100XP ultracentrifuge, Beckman Coulter). The supernatant and pellet were used as the soluble and particulate fractions of *S. iniae *cells, respectively [51]. In the second treatment procedure, the cellular fractions were obtained from *S. iniae *HD-1 by centrifugation using the protocol of Homonylo-McGavin & Lee [52,53]. Briefly, *S. iniae *HD-1 cells were grown overnight in 30 ml BHI and then washed by centrifugation at 4°C in a buffer composed of ice-cold 20 mM Tris and 1 mM MgCl_2 _(pH 7.0). The cell pellets were resuspended and incubated for 90 min in 0.3 ml of protoplast buffer (150 μl 60% raffinose (Beijing Newprobe Biotechnology Co., Ltd.), 15 μl 1 M Tris (pH 7.4), 6 μl 100 mM phenyl-methyl sulfonyl fluoride (MBchem, Inc.), 3 μl 1 M MgCl_2_, 15 μl 25,000 U ml^-1 ^mutanolysin (Sigma-Aldrich, Inc.), 15 μl 270,000 U ml^-1 ^lysozyme, and 96 μl ddH_2_O). The cell wall extracts were separated from the spheroplasts by centrifugation at 10,000 × *g *for 10 min. The pelleted protoplasts were washed, suspended in 2 ml PBS-sucrose buffer, and disrupted by sonication, as described above. The supernatant and pellet obtained after centrifugation at 248,000 × *g *for 1 h were used as the soluble and particulate fractions of the protoplasts, respectively. All cellular fractions were analyzed by western blotting using the rabbit anti-MtsA antibodies.

### Detection of the heme-binding activity of MtsA

The pyridine hemochrome assay [28] was used to analyze heme binding to MtsA. Purified MtsA in 750 μl of 10 mM Tris-HCl (pH 8.0) was mixed with 170 μl of pyridine (Sigma-Aldrich, Inc.), 75 μl of 1 N NaOH, and 2 mg of sodium hydrosulfite (Beijing Newprobe Biotechnology Co., Ltd.), and heme content was determined by measuring the absorbance (■, black square) at 418 nm with a UV-visible spectrophotometer (Uvmini-1240, Shimadzu). Purified catalase-peroxidase (KatG, Beijing Newprobe Biotechnology Co., Ltd.), a known heme-containing protein, was used as the positive control (Δ, white triangle) [54].

### Measurement of iron in MtsA by ICP-AES

The levels of Fe, Zn, Ca, Mg, and Mn in purified MtsA were determined by inductively coupled plasma-atomic emission spectrometry (ICP-AES) using an IRIS (HR) ICP-AES instrument [55]. Briefly, 0.1 g purified MtsA was immersed in 15 ml nitric acid in an electric cooker. After 3 h nitrification, 1 ml perchloric acid was added and treated for 1 h. The liquid was filter sterilized and analyzed by ICP-AES. A sample lacking purified MtsA was used as the negative control. To achieve contamination-deprivation conditions, all utensils were soaked overnight in nitric acid, and rinsed 6 times with ddH_2_O.

### Detection of anti-MtsA antibodies in sera from Kunming mice that were experimentally infected with *S. iniae *HD-1

To detect the presence of specific anti-MtsA antibodies in the sera from Kunming mice, 10 male Kunming mice (20 ± 2 g) were purchased from Guangdong Laboratory Animals Research Center, and approval from the Animal Ethics Committee of Life Sciences Institute was obtained prior to using the animals for research. The experiments were performed as stipulated by the China State Science and Technology Commission [47]. Mice were acclimatized at the SPF animal center and fed twice daily for 2 weeks in the laboratory of the Life Science Institute prior to use. Each mouse was injected with 100 μl of 6.2 × 10^8 ^CFU ml^-1 ^*S. iniae *HD-1 cells, and the infected sera were collected 10 days post infection. The infected sera and purified MtsA were used in dot-blot and western-blot assays. The sera from 10 Kunming mice injected with PBS were used as the negative control.

### Statistical analysis

The nucleotide and deduced amino acid homology analysis of *mtsABC *was carried out by ClustalX 1.83 and NCBI BLAST http://blast.ncbi.nlm.nih.gov/Blast.cgi. The presumed signal sequence was predicted by the signalP 3.0 Server http://www.cbs.dtu.dk/services/SignalP/. The theoretical pI/MW was analyzed by the ExPASy Compute pI/MW tool http://www.expasy.org/tools/pi_tool.html. The main domains of *mtsABC *were detected by the SMART software http://smart.embl-heidelberg.de/. The amino acid sequences were aligned using the SECentral Align Multi 4 program. To determine whether *mtsABC *is a Lipoprotein, its sequence was assessed by the ScanProsite analysis software http://www.expasy.ch/tools/scanprosite/. All statistical analyses were performed using the SPSS 16.0 software (SPSS Inc., USA).

## Authors' contributions

LLZ carried out the molecular genetic studies, participated in the sequence alignment studies, performed the statistical analysis, and drafted the manuscript. JW carried out the function studies and participated in the sequence alignment studies. HBF carried out the infection assay. MQX conceived of the study and participated in its design and coordination. AXL participated in the conceived of the study and helped to draft the manuscript. All authors read and approved the final manuscript.

## Supplementary Material

Additional file 1 **Tables 1-7**. Microsoft word file containing Tables 1-7 as individual tab-accessible tables within a single file (Supplemental Tables 1-7). Click here for file

Additional file 2 **Figures 1-4**. Microsoft word file containing Figures 1, 2, 3, 4 as individual tab-accessible figures within a single file (Supplemental Figures 1-4). Click here for file
