# Supplementary material for: A solute-binding protein for iron transport in Streptococcus iniae
Source: BMC Microbiol. 2010 Dec 1;10:309. doi: 10.1186/1471-2180-10-309 (PMC3014919; doi:10.1186/1471-2180-10-309)
Supplement: Additional file 1 — Tables 1-7. Microsoft word file containing Tables 1-7 as individual tab-accessible tables within a single file (Supplemental Tables 1-7). [file 1471-2180-10-309-S1.DOC]

**Additional File 1: TABLE 1: Proteins to which MtsABC have close identity and similarity**

| **Mts**  **protein** | **Protein name** | **Function** | **Organism** | **Identity/similarity**  **(%)** | **Length of amino acids compared** |
| --- | --- | --- | --- | --- | --- |
| **MtsA** | MtsA | Metal solute-binding lipoprotein | *Streptococcus dysgalactiae subsp. equisimilis* GGS_124 | 260/281 (92.5%) | 310 |
|  | PsaA | Metal solute-binding lipoprotein | *Streptococcus equi subsp. equi* 4047 | 262/283 (92.5%) | 310 |
|  | MtuA | Metal solute-binding lipoprotein | *Streptococcus uberis* 0140J | 258/282 (91.5%) | 310 |
|  | MtsA | Metal solute-binding lipoprotein | *Streptococcus pyogenes str. Manfredo* | 259/281 (92.2%) | 310 |
|  | MtsA | Metal solute-binding lipoprotein | *Streptococcus agalactiae* A909 | 237/276 (85.9%) | 309 |
|  | PsaA | Metal solute-binding lipoprotein | *Streptococcus oralis* ATCC 35037 | 223/272 (82.0%) | 310 |
|  | PsaA | Metal solute-binding lipoprotein | *Streptococcus parasanguinis* ATCC 15912 | 223/270 (82.6%) | 310 |
|  | PsaA | Metal solute-binding lipoprotein | *Streptococcus gallolyticus* UCN34 | 227/272 (83.5%) | 310 |
|  | PsaA | Metal solute-binding lipoprotein | *Streptococcus pneumoniae* TCH8431/19A | 223/269 (82.9%) | 310 |
| **MtsB** | MtsB | ATP-binding protein | *Streptococcus dysgalactiae subsp. equisimilis* GGS_124 | 161/202 (79.7%) | 239 |
|  | MtsB | ATP-binding protein | *Streptococcus equi subsp. zooepidemicus* MGCS10565 | 157/199 (78.9%) | 236 |
|  | MtsB | ATP-binding protein | *Streptococcus pyogenes str. Manfredo* | 160/198 (80.8%) | 239 |
|  | MtsB | ATP-binding protein | *Streptococcus uberis* 0140J | 149/189 (78.8%) | 240 |
|  | ZnuC | ATP-binding protein | *Lactococcus lactis subsp. cremoris* SK11 | 138/179 (77.1%) | 236 |
|  | MtsB | ATP-binding protein | *Streptococcus agalactiae* A909 | 139/180 (77.2%) | 236 |
| **MtsC** | MtsC | Transmembrane permease protein | *Streptococcus pyogenes str. Manfredo* | 235/262 (89.7%) | 282 |
|  | MtsC | Transmembrane permease protein | *Streptococcus dysgalactiae subsp. equisimilis* GGS_124 | 231/264 (87.5%) | 282 |
|  | MtsC | Transmembrane permease protein | *Streptococcus equi subsp. Zooepidemicus* MGCS1056*5* | 229/261 (87.7%) | 275 |
|  | MtsC | Transmembrane permease protein | *Streptococcus agalactiae* A909 | 220/250 (88.0%) | 270 |
|  | MtsC | Transmembrane permease protein | *Lactococcus lactis subsp. lactis* KF147 | 223/257 (86.8%) | 275 |

**Additional File 1: TABLE 2: The consensus residues of *mtsABC*** as ABC transporter class

| **Protein name** | **Domains** | **Protein residues** | **Conserved residues** |
| --- | --- | --- | --- |
| **MtsA** | Lipoprotein peptidase cleavage site | ITACS(18-22) | L(S/A)(A/G)C(S/G) |
| **MtsB** | TroA-like domain | 67-270^a^ | TroA domain |
|  | Walker site A | GPNGAGKST(35-43) | GxxGxGKS/T |
|  | Walker site B | YIFLDEPF(155-162) | hhhhDEPT |
|  | ABC signature | LSGGQFQR(135-138) | LSGGQQR/KQR |
|  | 4^th^ motif | VVHHD | hhhH^+^ |
| **MtsC** | TMs | Four TMs^b^ | TMs |

Conserved sequence motifs from MtsB for the ATP-domains of ABC transporter class as defined by Linton & Higgins. X, any residue; h, hydrophobic residue; +, charged residue. ^a^ HEYEPLPEDVEKTTNADLIFYNGINLEDGGQAWFTKLVKNAKKTKNKDYFAVSDGIDVIYLEGENEKGKEDPHAWLNLENGVIYSKNIA KQLMAKDPENKDYYQKNLDAYVAKLEKLDQEAKSAFDKIPDNKKVIVTSEGCFKYFSKAYKVPSAYIWEINTEEEGTPDQISSLIEKLKAKKPSALFVESSVDSRPMKSVSKDSG. ^b^ was predicted by the SMART software.

**Additional File 1: TABLE 3: Lipoprotein sequence patterns**

| **Pattern** | **Pattern expression** |
| --- | --- |
| G+LPP | <[MV]-X-[RK]-{DERKQ}-[LIVMFESTAG]-[LVIAM]-[IVMSTAFG]-[AG]-C |
| G+LPPv2 | <[MV]-X-[RK]-{DERK}-[LIVMFESTAGPC]-[LVIAMFTG]-[IVMSTAGCP]-[AGS]-C |
| PS51257 | {DERK}-[LIVMFWSTAG] -[LIVMFYSTAGCQ]-[AGS]-C |

‘<’indicates the pattern is restricted to the N-terminus and X is any amino acid.

**Additional File 1: TABLE 4:Mean anti-MtsA antibody titers as measured by dot blotting obtained from mice immunized with purified MtsB.**

| **Protein** | **Negative control** | **Antibody titer of 7 days after each round of immunization** | | | | |
| --- | --- | --- | --- | --- | --- | --- |
|  |  | **1^st^ round** | **2^nd^ round** | **3^rd^ round** | | **4^th^ round** |
| **MtsB** | ND | 1:4,800 | 1:22,000 | 1:48,000 | 1:50,000 | |

ND, not detected (<20).

**Additional File 1: TABLE 5: Detection of iron associated with purified** MtsA by ICP-AES

| **Metallic Elements** |  | **Content (mg L^-1^) *** |  |
| --- | --- | --- | --- |
|  | **1** | **2** | **3** |
| **Ca** | <0.006 | <0.006 | <0.006 |
| **Fe** | 1.43 | 1.38 | 1.33 |
| **Mg** | <0.006 | <0.006 | <0.006 |
| **Mn** | <0.006 | <0.006 | <0.006 |
| **Zn** | <0.006 | <0.006 | <0.006 |

* The contents of five metallic elements in 20 μM MtsA have been showed.

**Additional File 1: TABLE 6-1: Primer pairs for cloning *mtsABC* EST sequences of *S. iniae* HD-1**

| **Primer** | **Sequence （5′-3′）** | **Amplicon size（bp）** |
| --- | --- | --- |
| ***mtsA* F1** | TTTGGATGCCTATGTAGC | 355 |
| ***mtsA* R1** | ATCACCGTCTTGACCTTT | 355 |
| ***mtsA* F2** | TTAGACCAAGAAGCGAAAT | 237 |
| ***mtsA* R2** | TGGACGGCTATCAACACT | 237 |
| ***mtsB* F1** | TTAGCATTGAAGGTCCCG | 328 |
| ***mtsB* R1** | ATTGGCGCATCAGCATAG | 328 |
| ***mtsB* F2** | TTAGCATTGAAGGTCCCG | 167 |
| ***mtsB* R2** | TCGTTGCTCCACATAGGC | 167 |
| ***mtsC* F1** | GGCAGTAGGTTGTTTCAT | 414 |
| ***mtsC* R1** | TTAGCAAGCACTGGGTCA | 414 |
| ***mtsC* F2** | AAAATAGCGTGATTAAAGGG | 247 |
| ***mtsC* R2** | TTAGCAAGCACTGGGTCA | 247 |
| ***mtsAC* F1** | TTTGGATGCCTATGTAGC | 1674 |
| ***mtsAC* R1** | TTAGCAAGCACTGGGTCA | 1674 |
| ***mtsAC* F2** | TTAGACCAAGAAGCGAAAT | 1705 |
| ***mtsAC* R2** | TTAGCAAGCACTGGGTCA | 1705 |

*S. iniae* HD-1 *mtsABC* has three genes, each has two primer pairs for cloning EST. F1, R1 prefer to outer primer pairs for nested-PCR; F2, R2 prefer to inner primer pairs for nested-PCR.

**Additional File 1: TABLE 6-2: Specific primer pairs for siteFinding-PCR**

| **Primer** | **GSP1 Sequence （5′-3′）** | **GSP2 Sequence （5′-3′）** | **GSP3 Sequence （5′-3′）** |
| --- | --- | --- | --- |
| ***mtsABC* UP** | AAGCTGACGGCACTTTGTAAGC | CAACCATGCGTGTGGATCTTC | CAACATCTTCTGGCAACGGCTC |
| ***mtsABC* DOWN** | TTGGGAATATTCTGGCGGTTC | GCTAAGTCAATGGGGTATCGAGTTGG | CACACCAGCTGCAACAGCCTATC |

**Additional File 1: TABLE 6-3: Sequences of two SiteFinders and their primers (SFP1 and SFP2) for siteFinding-PCR**

| **Primer** | **Sequence （5′-3′）** |
| --- | --- |
| **SFP1** | CACGACACGCTACTCAACAC |
| **SFP2** | ACTCAACACACCACCTCGCACAGC |
| **SiteFinder-1** | CACGACACGCTACTCAACACACCACCTCGCACAGCGTCCTCAAGCGGCCGCNNNNNNGCCT |
| **SiteFinder-2** | CACGACACGCTACTCAACACACCACCTCGCACAGCGTCCTCAAGCGGCCGCNNNNNNGCGC |

**Additional File 1: TABLE 7: Primer pairs used in reverse transcriptase-PCR of *mtsABC***

| **Primer** | **Sequence (5′-3′)** | **Amplicon size (bp)** |
| --- | --- | --- |
| ***mtsA* F** | ATGTTTAAAAAAATAAGCCTAGC | 930 bp |
| ***mtsA* R** | TTATTTTGCTAAGCCTTCTGAA | 930 bp |
| ***mtsB* F** | ATGATTAAAACAAAAAATTTGTCTG | 729 bp |
| ***mtsB* R** | TTAACATGCTTTTTCTCCTTTT | 729 bp |
| ***mtsC* F** | ATGTTAATGAAGTTTATTGATGGTT | 852 bp |
| ***mtsC* R** | TTAATGTCTATTTTTACTAACTAG | 852 bp |
| ***mtsAB* F** | ATGTTTAAAAAAATAAGCCTAGC | 1724 bp |
| ***mtsAB* R** | TTAACATGCTTTTTCTCCTTTT | 1724bp |
| ***mtsBC* F** | ATGATTAAAACAAAAAATTTGTCTG | 1574 bp |
| ***mtsBC* R** | TTAATGTCTATTTTTACTAACTAG | 1574 bp |
