# Supplementary material for: A solute-binding protein for iron transport in Streptococcus iniae
Source: BMC Microbiol. 2010 Dec 1;10:309. doi: 10.1186/1471-2180-10-309 (PMC3014919; doi:10.1186/1471-2180-10-309)
Supplement: Additional file 2 — Figures 1-4. Microsoft word file containing Figures 1, 2, 3, 4 as individual tab-accessible figures within a single file (Supplemental Figures 1-4). [file 1471-2180-10-309-S2.DOC]

In this paper, *mtsA* gene was cloned and the PCR product was isolated from the plasmid after a double digestion with restriction enzymes *Bam*HI and *Xho*I, and ligated into the compatible site of pET-32a-c (+) Vector to yield recombinant protein MtsA. The recombinant protein MtsA has a tag from Trx·Tag to *EcoR V* (Fig. 1), which has a molecular weight of 17.7-kDa.


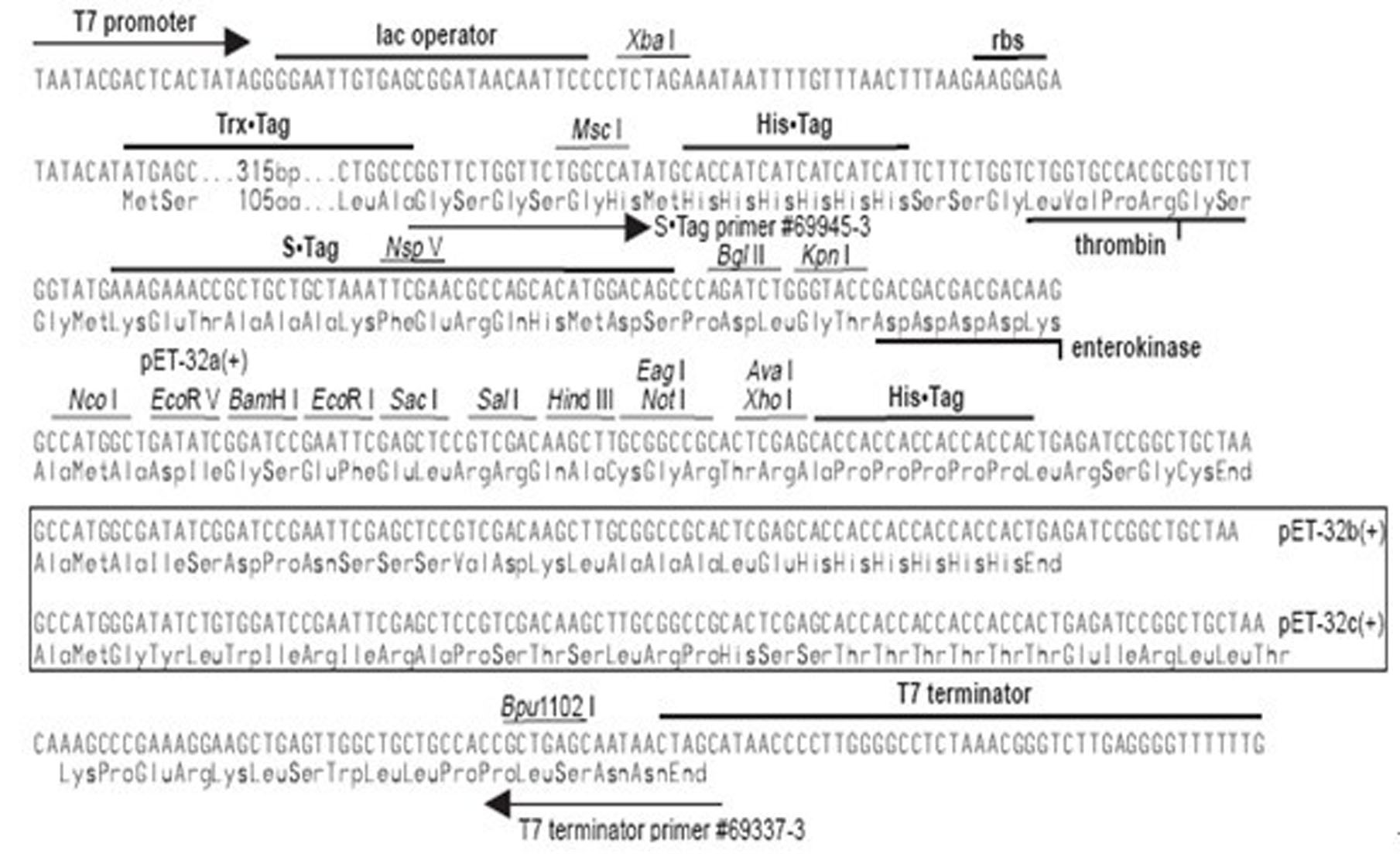


Fig. 1 pET-32a-c(+) Vector Multiple Cloning Sequences

The following sequences (Fig. 2) are the recombinant protein of MtsA (with tag, Theoretical pI/Mw: 5.41 / 49472.98), and the sequences of aim protein were underlined (without tag, Theoretical pI/Mw: 5.48 / 31770.00).

MSDKIIHLTDDSFDTDVLKADGAILVDFWAEWCGPCKMIAPILDEIADEYQGKLTVAKLNIDQNPGTAPKYGIRGIPTLLLFKNGEVAATKVGALSKGQLKEFLDANLAGSGSGHMHHHHHHSSGLVPRGSGMKETAAAKFERQHMDSPDLGTDDDDKAMADIGSASKDKKLDVVVTNSIIADMTKNIAGKKINLHSIVPIGQDPHEYEPLPEDVEKTTNADLIFYNGINLEDGGQAWFTKLVKNAKKTKNKDYFAVSDGIDVIYLEGENEKGKEDPHAWLNLENGVIYSKNIAKQLMAKDPENKDYYQKNLDAYVAKLEKLDQEAKSAFDKIPDNKKVIVTSEGCFKYFSKAYKVPSAYIWEINTEEEGTPDQISSLIEKLKAKKPSALFVESSVDSRPMKSVSKDSGIPIYSEIFTDSVAKKGQDGDSYYAMMKWNLDKISEGLAK

Fig. 2 The Sequences of Recombinant Protein MtsA

To confirm the specificity of MtsA, we expressed and purified the pET-32a-c (+) Vector without *mtsA* that has the molecular weight of about 22.0-kDa (Fig. 3). The purified protein was incubated with infected sera from Kunming mice with *S. iniae* HD-1 infection for 2 h, and the blocked sera were further used in western blotting with purified MtsA. The results of western blotting presented that the peptide fragments were detected when the blocked antibodies were used in the experiment (Fig. 4). This result showed that the vector tag of recombinant protein MtsA did not affect the specificity of MtsA.


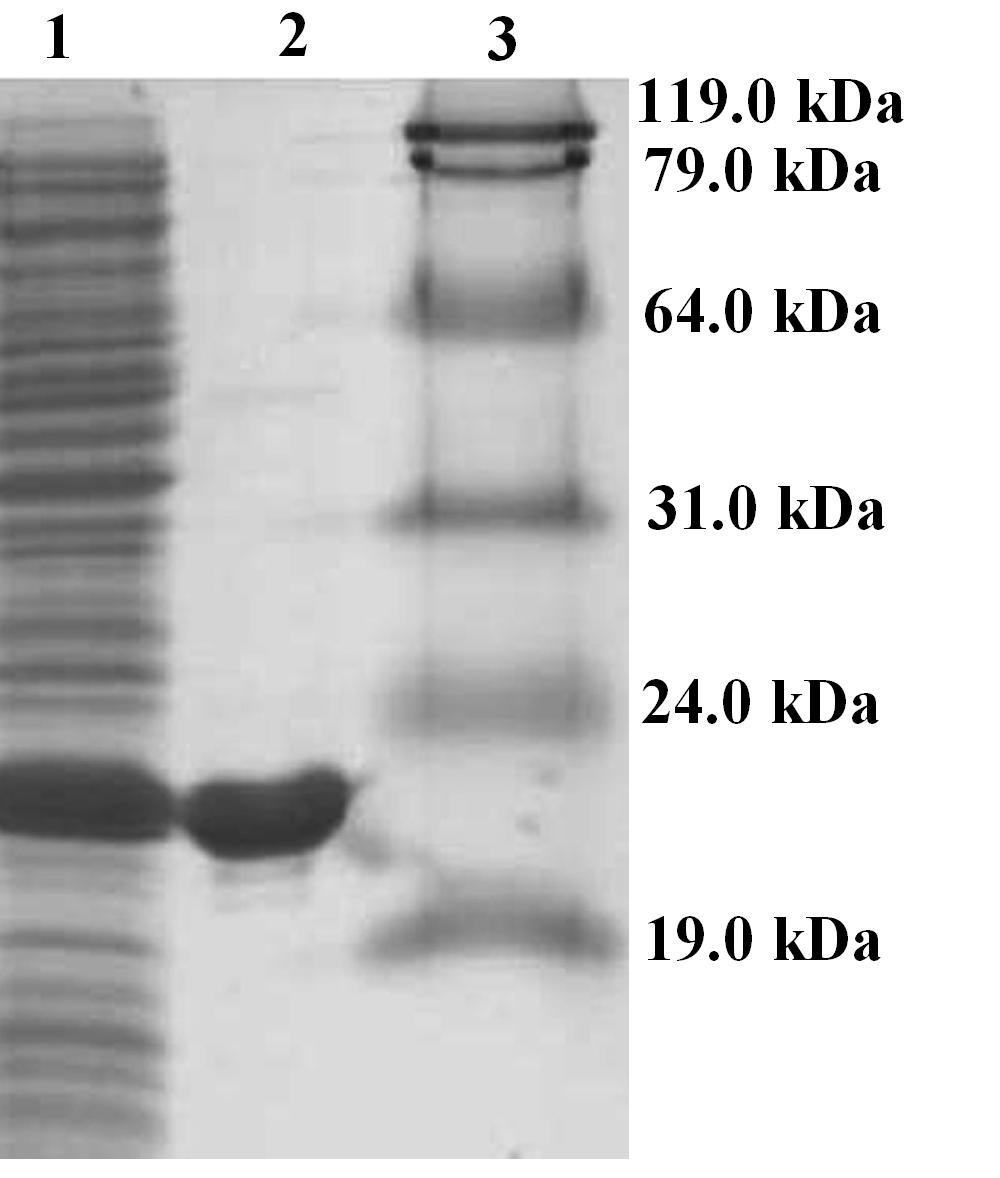


Fig. 3 Production and Purification of Recombinant pET-32a-c (+) Vector

1: The total lysates with IPTG; 2: Purified pET-32a-c (+) Vector; 3: The molecular marker

**
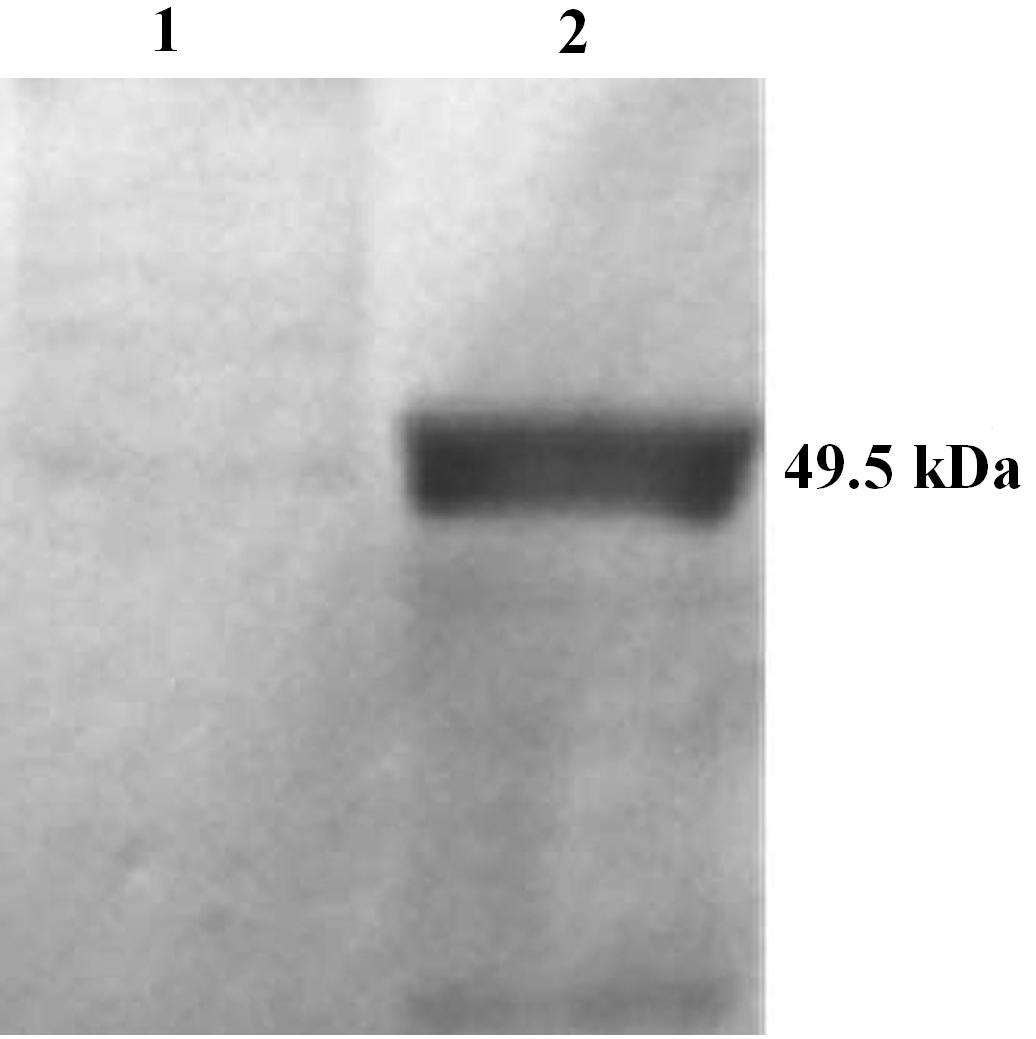
**

Fig. 4 Western Blotting Analysis of MtsA with Blocked Antibodies

1: Western blotting results of control sera; 2: Western blotting results of infected sera
